# Highlight selection of radiochemistry and radiopharmacy developments by editorial board

**DOI:** 10.1186/s41181-021-00128-x

**Published:** 2021-03-18

**Authors:** Silvio Aime, Mohammed Al-Qahtani, Martin Behe, Guy Bormans, Giuseppe Carlucci, Jean N. DaSilva, Clemens Decristoforo, Adriano Duatti, Philip H. Elsinga, Klaus Kopka, Xiang-Guo Li, Zhibo Liu, Robert H. Mach, Oskar Middel, Jan Passchier, Marianne Patt, Ivan Penuelas, Ana Rey, Peter J. H. Scott, Sergio Todde, Jun Toyohara, Danielle Vugts, Zhi Yang

**Affiliations:** 1 University of Torino, Torino, Italy; 2 King Faisal Specialist Hospital and Research Center, Riyadh, Saudi Arabia; 3 Paul Scherrer Institute, Villigen, Switzerland; 4 Katholieke Universiteit Leuven, Leuven, Belgium; 5 UCLA Molecular and Medical Pharmacology Department, Los Angeles, USA; 6 University of Montreal, Montreal, Canada; 7 Universitaetsklinikum fur Nuclearmedizin, Innsbruck, Austria; 8 University of Ferrara, Ferrara, Italy; 9 University Medical Center Groningen, University of Groningen, Groningen, The Netherlands; 10 Helmholtz-Zentrum Dresden-Rossendorf (HZDR), Dresden, Germany; 11 Turku PET-center, Turku, Finland; 12 Peking University, Beijing, China; 13 University of Pennsylvania, Philadelphia, USA; 14 St. Olavs Hospital, Trondheim, Norway; 15 Invicro, London, UK; 16 University of Leipzig, Leipzig, Germany; 17 University Clinic of Navarra, Pamplona, Spain; 18 Universidad de la Republica, Montevideo, Uruguay; 19 University of Michigan, Ann Arbor, USA; 20 University of Milano-Bicoccia, Milan, Italy; 21 Tokyo Metropolitan Institute of Gerontology, Tokyo, Japan; 22 Amsterdam UMC, Amsterdam, The Netherlands; 23 Peking University Cancer Hospital, Beijing, China

## Abstract

**Background:**

The Editorial Board of EJNMMI Radiopharmacy and Chemistry releases a biyearly highlight commentary to update the readership on trends in the field of radiopharmaceutical development.

**Results:**

This commentary of highlights has resulted in 23 different topics selected by each member of the Editorial Board addressing a variety of aspects ranging from novel radiochemistry to first in man application of novel radiopharmaceuticals.

**Conclusion:**

Trends in radiochemistry and radiopharmacy are highlighted demonstrating the progress in the research field being the scope of EJNMMI Radiopharmacy and Chemistry.

## Introduction

Each individual member of the Editorial Board has selected a highlight article that has appeared in the radiochemistry and radiopharmacy literature during the period July 2020–January 2021. The aim of this collaborative initiative is to create a biyearly overview for the readers summarizing the latest trends in the field.

## Design and gallium-68 labelling of pH-triggered aggregation of melanin nanoparticles for enhanced accumulation in tumors

### By Ivan Penuelas

Melanin is an endogenous pigment that is distributed widely throughout human tissues, thus making it potentially safe for in vivo application. Moreover, melanin can load chemotherapeutic drugs with aromatic structures via π-π stacking and/or hydrogen bonding, and the drug release can be stimulated by multiple methods, including near infrared light, pH, and reactive oxygen species.

Melanin includes abundant carboxyl groups, amino groups, and phenolic hydroxyl groups, and could thus be seen as a natural multisite metal chelating agent, making it capable of complexing many metal ions (including several radiometals) under mild conditions.

Melanin can also be incorporated into nanoparticles with controllable sizes from a few nanometers to hundreds of nanometers. While small nanoparticles (< 20 nm) can avoid macrophage recognition and penetrate tissues more deeply when they reach the tumor site, they continue to backflow into the bloodstream or are cleared into the surrounding tissues, decreasing retention within the tumor. On the other hand, nanoparticles of about 100 nm in size have good retention but still high accumulation in the liver and pancreas before reaching the tumor, resulting in relatively low drug concentrations at the tumor site and potential increased toxicity.

**Fig. 1:**
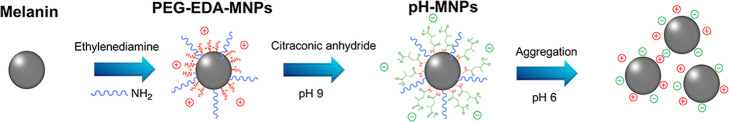
Schematic illustration of the preparation process of the pH-MNPs. (Reproduced from Liu Q, Fang H, Gai Y, Lan X. pH-Triggered Assembly of Natural Melanin Nanoparticles for Enhanced PET Imaging. Front Chem. 2020;8:755)

Liu et al. (2020) prepared small melanin nanoparticles (MNP) decorated with PEG2000 and ethylenediamine to provide multiple amine groups in the surface that where then reacted with citraconic anhydride to form amide bonds (Fig. 1). MNP had ≈12 nm and positive Z potential. Radiolabelling with gallium-68 was performed at pH = 7 with low-moderate yield, but after PD10 purification high purity ^68^Ga-MNP was produced.

At acidic pH, as commonly found in the tumor microenvironment due to hypoxia, the citraconic acid tends to hydrolyze abruptly thus generating multiple positive and negative charges in the surface of the nanoparticles. Such charge modification, induced nanoparticle aggregation producing > 100 nm aggregates. MicroPET imaging of tumor bearing mice showed specific accumulation of pH-MNPs in the tumors even at short times (tumor to muscle ratios increased from 4 to 29 between 1 and 3 h).

Other authors have recently radiolabelled small PEG-MNP with copper-64 as a potential theragnostic agent (Zhou et al., 2020).

### Multifunctional molecular imaging probes: a step forward for in vivo biochemistry studies

#### By Ana Rey

As an alternative approach for the design of multimodal imaging tracers, a series of 2 + 1 mixed ligand luminescent Re(I) tricarbonyl complexes and their isostructural ^99m^Tc congeners were prepared and evaluated as optical-nuclear probes (Slikboer et al., 2020). The complexes incorporate simultaneously a bidentate phenyl imidazole-fused phenanthroline ligand and N-methylimidazole as monodentate coligand. These types of complexes have attractive optical properties including broad absorbance extending into the visible spectrum, and also emission spectra extending into the near-infrared region.

**Fig. 2:**
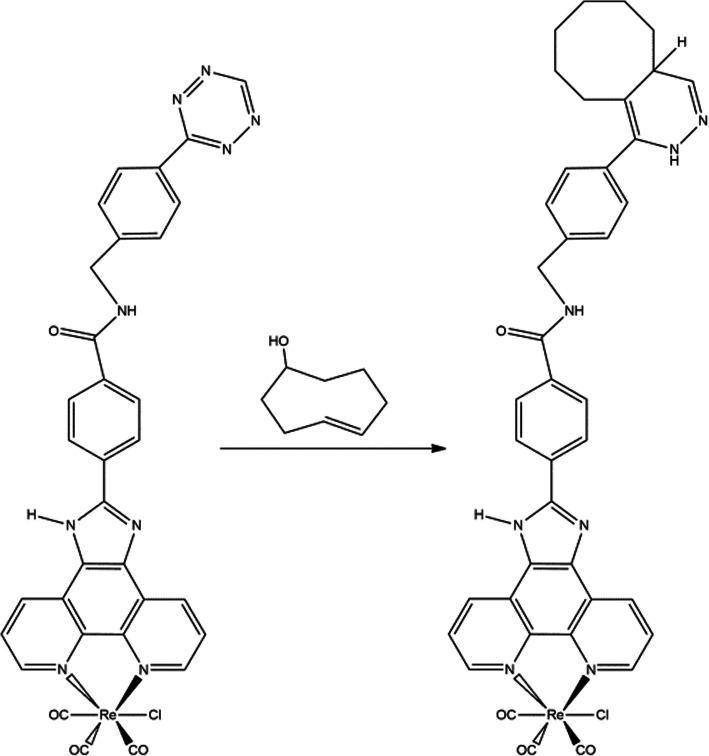
Synthesis of click-based Re(I)-tricarbonyl complex

Both the monodentate imidazole and the bidentate phenantroline can be derivatized to introduce a targeting moiety for molecular imaging. In this case the authors used the reaction of a carboxylic acid with an amino group for bioconjugation of a tetrazine moiety, which can be used for pretargeting strategies by the well-known bioorthogonal reaction between tetrazine and trans-cyclooctene (Fig. 2). An additional interesting aspect of this approach is the fact that the heterocycle of the tetrazine can quench the luminescence of the Re-complex, a phenomenon that is reversed by the trans-cyclooctene, generating a sort of turn-on type probe.

### Tetrabutylammonium tosylate as inert phase-transfer catalyst: the key to high efficiency S_N_2 radiofluorinations

#### By Mohammed Al Qahtani

**Fig. 3:**
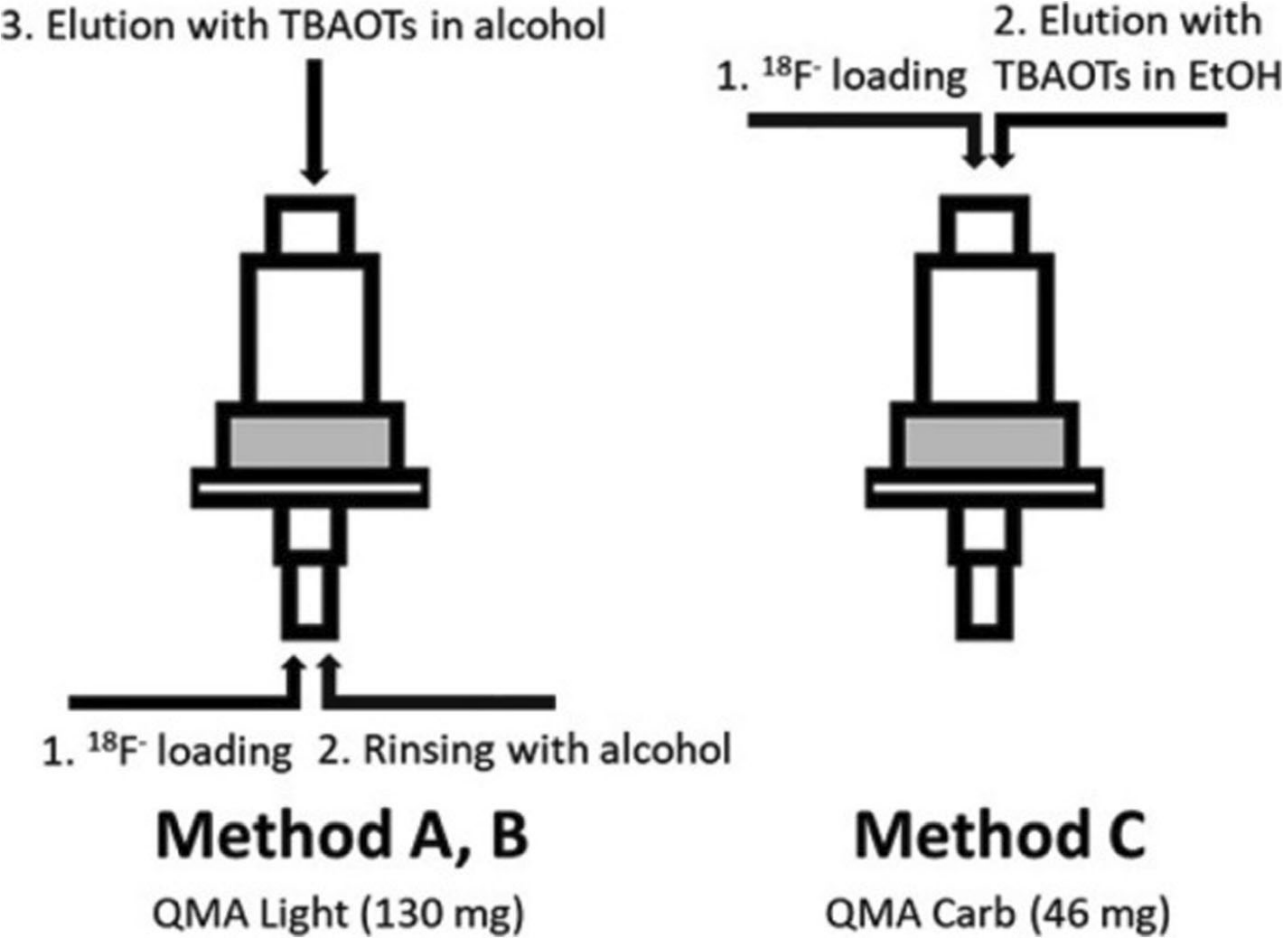
The schematic presentation of loading-elution protocols used in this study. (With permission from Orlovskaya et al., 2020 by Copyright Clearance Center’s Rightslink)

Tetrabutylammonium tosylate (TBAOTs) is described as an inert phase-transfer catalyst (PTC) for aliphatic S_N_2 radiofluorinations with some examples of commonly used fluorine-18 radiotracers (Orlovskaya et al., 2020). A unique methodology is presented clearly pointing out its advantages for the ^18^F-fluorinations as they show downscaling quantities of both TBAOTs and labeling precursors for [^18^F]fluoro-2-deoxy-glucose (FDG), [^18^F]fluoromidomidazole (FMISO), [^18^F]fluoroethyl tyrosine (FET), [^18^F]fluoro-L-thymidine (FLT), 16α-[^18^F]fluoro-17β-estradiol ([^18^F]FES) and radiolabeling synthon, 2-[^18^F]fluoroethyl tosylate ([^18^F]FEOTs). In short, non-aqueous solutions of TBAOTs containing very low amounts of the PTC (1 mg) allowed for efficient ^18^F-recovery from commercially available anion exchange cartrdges. The novel protocol produced highly reactive [^18^F]fluoride without the need for azeotropic drying. High radiochemical conversions (RCC 70–95%) were achieved using significantly reduced amounts of aliphatic substrates compared to traditional approaches. By reducing the precursor amounts by 5–10 orders of magnitude, sources of chemical impurities in the final formulations are minimized, allowing for simplification of the purification procedures. The overall process developed compares favorably with previously reported methods due to circumvention of the time consuming azeotropic evaporation and reduction in consumption of expensive precursors (Fig. 3). A novel sorption-elution protocol was demonstrated suitable for automation by its implementation on the GE TRACERlab FX-N Pro synthesis module. These findings will be of use in developing robust and scalable syntheses of various PET radiotracers using different automation platforms.

### ^18^F-Labelled nicotinic acid, a prosthetic agent with simplified radiosynthesis

#### By Xiang-Guo Li

To radiolabel biomolecules like peptides, a radiolabeled prosthetic agent is frequently needed. *N*-Succinimidyl 4-[^18^F]fluorobenzoate ([^18^F]SFB) has proved useful as a prosthetic agent for preclinical as well as clinical applications with PET. However, the radiosynthesis of [^18^F]SFB is relatively complicated to set up in the setting of GMP. A few research groups have reported analogues of SFB, with the aim for simplifying radiosynthesis protocols. Recently, a “fluorination on the Sep-Pak” method to prepare a prosthetic agent nicotinic acid *N-*hydroxysuccinimide ester (6-[^18^F]SFPy) (Basuli et al., 2020) was published. The actual ^18^F-fluorination takes place when passing the precursor compound through a solid-phase extraction cartridge pre-loaded with [^18^F]fluoride. Because of the simplicity of the procedure, an automated radiosynthesis has been set up for this work.

### Cyclotron production of radiometals in liquid targets may be worthwhile

#### By Sergio Todde

Cyclotron production of radiometals in the form of aqueous solutions has long been considered very attractive, due to the ease of handling liquid vs solid targets. Major hurdles to be overcome were very high “in-target” pressures even at low beam current, salt precipitation and low production yield. Since the early attempts, efficiency has constantly improved. In-target pressure was controlled using nitrate salts of the enriched isotopes dissolved in diluted nitric acid as starting materials, that promote free radical recombination and prevent gas formation. Salt precipitation may be prevented by controlling the concentration of the irradiated solution, while production yields may be improved by increasing beam current and time, although the author’s findings (Pandey and De Grado, 2020) show that this may work only if the concentration of nitric acid is simultaneously increased, and when target cooling is very efficient. Results related to the production of gallium-68, yttrium-86, zirconium-89, and zinc-63 are reported. As radionuclide “in-target” growth is an inverse function of its half-life, liquid target radiometal production may be advantageous for short-lived radionuclides, such as gallium-68, but less useful in case of e.g. zirconium-89. Gallium-68 is indeed the currently most important radionuclide for which the cyclotron production with liquid targets may be of interest (Rodnick et al., 2020). Up to 10 GBq of the radionuclide were obtained after irradiating a [^68^Zn]Zn(NO_3_)_2_ target for 60 min at 40 μA, but there is room for further improvements. However, useful amounts for internal clinical/pre-clinical research were also obtained for the other concerned radioisotopes.

### High yield cyclotron production of a novel ^133/135^La theranostic pair for nuclear medicine

#### By Oskar Middel

A scientific report (Nelson et al., 2020) appeared on the study of cyclotron targetry developments, and the fine-tuning of the existing ^132/135^La theranostic pair in costs and performance. The presented novel ^133/135^La theranostic pair and the discussion around where it fits in, related to other isotopes and theranostic pairs, must be a highlight for interested readers.

^133^La is superior to ^132^La in performance when comparing ^132/135^La and ^133/135^La pairs. The accessibility of cyclotron production using ^nat^Ba metal results in improved isotope yields and the lower branching ratio for ^133^La (7,2% compared to 41,2%) make it a more interesting isotope to use. The lower maximum positron energy of ^133^La (1,02 MeV compared to 3,67 MeV) would result in a much higher PET imaging spatial resolution.

Consequently, the ^133/135^La theranostic pair should allow more accurate imaging to track the treatment of small tumours and metastasis. This work facilitates future developments of this pair in a hospital setting. As the authors state, ^133^La has imaging properties comparable to ^11^C, but has a much longer half-life time (3,91 h). It is exciting how and if these developments are interpreted and picked up by others.

### Automated production enables reliable GMP-compliant supply of the ^18^F-somatostatin receptor ligand [^18^F]SiTATE

#### By Marianne Patt

With the development of ^18^F-labelled ligands for diagnosis of neuroendocrine tumors (NETs) modern nuclear medicine has a valuable tool at hand that is suitable to overcome the limitations of the corresponding ^68^Ga-tracers such as high costs and few patient doses per batch production. One of the most promising ligands for diagnosis of NETs by PET is [^18^F]SiTATE that was formerly named [^18^F]SiFA*lin*-TATE because of its dimethylated nitrogen structure which results in a quarternary amine similar to the cho*lin*e structure.

For [^18^F]SiTATE a highly efficient labelling procedure (RCY 54%) is described based on the silicon-fluoride acceptor (SiFA) technology using an automated cassette system (Lindner et al., 2020). Though the method is based on isotopic exchange of ^18^F for ^19^F, the product is obtained with high molar activity since only very low amounts of the corresponding ^19^F-precursor are required. By the nature of the labelling step almost no by-products are formed and therefore purification can be realized by a simple cartridge method instead of semipreparative HPLC. The short time required for the labelling reaction (5 min) and the application of a cartridge method for drying of the starting activity [^18^F]fluoride as well as simple purification of the final product contribute to an appealing overall synthesis time of 22 min only. Description of GMP-compliant quality control methods and exemplary clinical results complete this valuable article on state of the art radiopharmaceutical applications.

### Better biological understanding of targeted radionuclide therapy with modern biological methods

#### By Martin Behe

Targeted radionuclide therapy (TRT) becomes more and more common in clinical applications and shows promising results in cancer patients. Nevertheless, in some cases, TRT cannot destroy all cancer cells leading to relapse and therapy failure. Despite previous studies, the radio-resistance mechanism in cancer cells is still not fully understood. A better understanding of the activation of radioresistance mechanisms would help to develop more effective combinatory TRT. Stuparu et al. (2020) examined the TRT-triggered cell signaling in a prostate cancer mouse model treated with [^225^Ac]Ac- and [^177^Lu]Lu-PSMA-617 with proteomics and phosphoproteomics. They identified signaling alterations of genotoxic stress response pathways, including deregulation of DNA damage/replication stress response, TP53 protein, androgen receptor, PI3K/AKT and Myc signaling. They evaluated the role of TP53 in TRT by comparing the therapy effectiveness in a prostate cancer model with TP53 knockout compared to the wild type tumors. They could show as expected from the (phospho-)proteomics data that the TP53-knockout tumors are less sensitive to TRT. In the next step they will evaluate combinatory therapeutic approaches to block the initiated escape mechanism of the cells after TRT.

These results will allow evaluating a combinatory treatment, which aims at inhibition of these resistance mechanisms and may lead to enhanced treatment efficacy in cancer patients. The use of up-to-date biological methods to improve our understanding of the biological processes are important steps towards development of more effective TRT.

### A novel FAP-targeted imaging and treatment of mCRPC

#### By Giuseppe Carlucci

Fibroblast activation protein alpha (FAP) is a transmembrane serine protease expressed by cancer-associated fibroblasts (CAFs) in the microenvironment of epithelial tumors. FAP overexpression is associated with higher risk of tumor invasion, lymph node metastasis, and decreased overall survival. The localization of FAP to the tumor microenvironment and its association with aggressive phenotypes make it an ideal target for theranostic development. Although FAP expression was observed in multiple solid cancers, its presence in metastatic castration-resistant prostate cancer (mCRPC) is still poorly characterized. Therefore, there remains a need for a selective and sensitive imaging probe that can be widely used in patients with mCRPC.

As a target for the imaging and treatment of mCRPC FAP was introduced and the development of [^89^Zr]Zr-B12 IgG was described (Hintz et al., 2020). The authors outline the excellent selectivity and sensitivity of this probe toward FAP-expressing cells in several preclinical models of prostate cancer. In addition, they introduce a clinically relevant model of the prostate tumor microenvironment that could facilitate the development and translation of FAP-targeted agents for mCRPC.

Injection of [^89^Zr]Zr-B12 IgG resulted in high tumor uptake and retention compared to tumors administered with the [^89^Zr]Zr-isotype IgG control. The authors highlight that this molecule could be an ideal candidate for noninvasive PET/CT imaging in mCRPC, and more importantly, it represents a platform technology for the development of theranostics targeting FAP. The findings define that targeting FAP could be a successful strategy to develop novel imaging and therapeutic agents that are urgently needed in mCRPC.

### Shining a light on carbon-11 radiochemistry

#### By Robert H. Mach

The past decade has witnessed significant advances in “late stage” radiolabeling methods that can be applied to the synthesis of PET radiotracers. Most of these advances have focused on fluorine-18, whereas methods for incorporating carbon-11 via carbon-carbon bond formation are limited due to the harsh reaction conditions required by traditional C–C cross-coupling reactions (i.e. Stille, Suzuki, and Negishi couplings). Recently, the incorporation of a radiolabeled methyl group into either an *aromatic or aliphatic* carbon atoms using a novel metallophotoredox-catalyzed method was reported. The motivation for this research was driven by the high percentage of small molecule therapeutics that possess one or more methyl groups attached to another carbon atom (> 65% as of 2018), and the need to develop efficient means for preparing either tritium or carbon-11 isotopologues of pharmaceutically-relevant small-molecules. The resulting tritium labeled ligand may then be utilized for in vitro binding studies that are meant to aid in the interpretation of parallel carbon-11 PET imaging studies. This approach complements commonly used strategies that typically employ nucleophilic substituents on precursors such as phenols and anilines as labeling positions in S_N_2-type reactions.

The authors (Pipal et al., 2020) initially optimized the radiolabeling conditions using a [CT_3_]methyl-1-naphthalene-sulfonate, a stable and non-volatile synthon for CT_3_-methylations. They were able to demonstrate high radiolabeling yields in a variety of substrates, including both aromatic and aliphatic C–C coupling reactions. Once the reaction conditions were established for CT_3_-methylations, the authors focused on carbon-11 labeling methods using [^11^C]methyl iodide. The conditions described within the manuscript are general to a large number of functional groups, including ones typically involved in carbon-11 labeling reactions such as aromatic phenols. The authors showcase this capability by preparing [^11^C]PHNO in a single step without the use of protecting groups. An important observation was that the pseudo-first order kinetics of ^11^C-radiochemistry resulted in a much faster rate of incorporation relative to the CT_3_-radiomethylations (~ 5 min for carbon-11 versus 8 h for tritium). It was also demonstrated that the light source can be easily configured in commercially-available automated carbon-11 chemistry modules, which will enable the use of this radiosynthetic method to virtually every PET radiochemistry program currently utilizing [^11^C]MeI modules. This method should lead to an important breakthrough in the use of carbon-11 radiotracers in PET imaging studies and further streamline the radiosynthesis of high molar activity tritium compounds for use in in vitro binding studies.

### Another step towards microbe specific PET imaging of infections

#### By Clemens Decristoforo

Increasing efforts are being made to develop microbe specific radiopharmaceuticals based on metabolic processing for a more sensitive and specific diagnosis of infections with PET. These include sugar-alcohols like [^18^F]fluoro-deoxy-sorbitol, ^18^F-maltose derivatives, ^11^C-labelled-D-amino acids, radiolabelled siderophores or derivatives of p-aminobenzoic acid (PABA). 2-[^18^F]Fluoro-PABA was reported to target clinically important *Staphylococcus aureus* (*S.aureus*) infections with excellent sensitivity and specificity, however being rapidly metabolized and excreted, leading to impaired targeting efficiency. Li and co-workers now replaced the amine of 2-fluoro-PABA by a nitro group and the prepared the ethyl-ester thereby producing the pro-drug ethyl 2-[^18^F]fluoro-4-nitrobenzoate (2-[^18^F]F-ENB) to overcome these limitations (Li et al., 2020). In nicely designed in vitro studies they could show the hydrolysis of the ester and the specific conversion of the nitro group to the amine by a *S. aureus*-specific nitroreductase. In vivo results confirmed the specificity of 2-[^18^F]F-ENB to target *S. aureus* with excellent (17-fold) contrast between the sites of infection and sterile inflammation. Finally radiosynthesis of 2-[^18^F]F-ENB was optimized and adopted for a standard automated module with good radiochemical yields to allow clinical translation. This work is an excellent example of elegant tracer optimization and characterization, it adds to the increasing armory of metabolic PET tracers specific for infectious pathogens. We now can wait to see which of these promising PET-tracers will finally succeed in a clinical setting, such recent developments give hope that we will see an increasing role of PET in infection imaging based on highly specific radiopharmaceuticals.

### The evolution of ^18^F-chemistry@one must-have molecule

#### By Klaus Kopka

The paper “Nucleophilic Synthesis of 6-l-[^18^F]FDOPA. Is Copper-Mediated Radiofluorination the Answer?” published by Raisa N. Krasikova is one of the master examples of what we intend to do in radiochemistry and radiopharmacy: facilitating our lifes in the clinical radiochemistry environment. She chose a molecule of utmost clinical relevance for the diagnosis of different diseases, such as neurological disorders and cancer (Krasikova, 2020). However, until now the radiofluorination methods yielding [^18^F]FDOPA in satisfying activity amounts by fully automated radiosynthesis are still not optimal. Offering higher activity amounts to the clinic would result in higher clinical demands, as is the case for [^18^F]FDG. What makes the difference compared to [^18^F]FDG is the task to introduce radiofluorine into a non-activated aromatic system. Because of its feasible setup on automated radiosynthesizers many PET radiopharmacies nowadays still are using the electrophilic ^18^F-fluorodemetallation route using organotin precursors. The main disadvantages are not easy to handle [^18^F]F_2_ cyclotron targets, low RCYs and resulting low molar activities. So, over the years many radiochemistry groups tried hard to introduce radiofluorine into the aryl ring via the nucleophilic route. The evolution of introducing radiofluorine into the must-have molecule [^18^F]FDOPA via the nucleophilc route, starting with the early introduction of radiofluorine into the molecule with the necessary following chiral alkylation step, and ending with the Cu-mediated late-stage radiofluorination methods [including the minimalist approach] of iodonium salts, arylpinacol boronates and arylstannanes which resulted in significant potential for implementation in clinical productions, is highlighted. Currently the yields via Cu-mediated radiofluorination of trimethyl stannylated DOPA derivatives are already comparable with that of the cassette-based nucleophilic multistep radiosynthesis of 6-l-[^18^F]FDOPA optimized over years on the AllinOne, Trasis module, in which a chiral phase transfer catalyst and O’Donnel glycine substrate are used. It was finally concluded that radiofluorination of organostannanes in combination with azeotropic-drying free protocols is advantageous for automation, and at the same time labeling substrates that can be simply prepared from commercially available stannylated precursors are easily accessible. This should accelerate implementation of this [^18^F]FDOPA synthesis method on a variety of radiosynthesizers, giving a start into simplified routine GMP-compliant production. The main questions remains: Is the evolution of ^18^F-chemistry on this must-have molecule really finished?

### Scandium labelled bombesin antagonists are promising

#### By Philip H. Elsinga

Scandium radionuclides ^43^Sc and ^44g^Sc are new PET-radionuclides with half-lives of 3.89 h and 3.97 h, respectively and their availability is increasing by newly developed cyclotron production methods. Their half-lives are comparable to ^99m^Tc and therefore these scandium radionuclides may be used as alternative for ^99m^Tc as PET-equivalent. To further investigate the application of the mentioned scandium radionuclides, bombesin derivatives were prepared (Ferguson et al., 2020). Their binding affinity, biodistribution and tumor uptake of ^68^Ga (as gold standard) and ^44g^Sc-labelled DOTA-Ava-BBN2 were compared. These BBN2-peptides bind to the Gastrin Releasing Peptide Receptor (GRPR) which is overexpressed in several tumors including prostate and breast tumors. Both tracers were investigated in mice bearing PC3 and MCF7 xenografts. Binding affinity to GRPR of the ^44g^Sc-DOTA-Ava-BBN2 was 3-fold higher that the ^68^Ga-analog, but in vivo, both tracers displayed comparable biodistribution and tumor uptake with rapid blood and renal clearance in the 2 tumors animal models. Therefore, it is concluded that the scandium labelled BB2 tracer having excellent imaging characteristics should be further investigated. The focus should be switched to ^43^Sc as clinical use of ^44g^Sc may be limited because of high energy of 1157 keV gamma radiation, whereas ^43^Sc emits only a low energy gamma of 372 keV.

### A rapid ^18^F–^19^F isotopic exchange radiolabeling method of β-diketone molecules for PET/fluorescence imaging

#### By Jean N. DaSilva

**Fig. 4:**
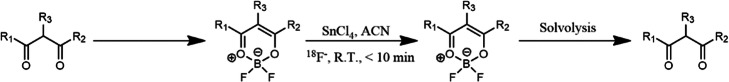
Difluoro-dioxaborinins synthesis, radiosynthesis and solvolysis. Reprinted (adapted) with permission from An F, Nurili F, Sayman H, Ozer Z, Cakiroglu H, Aras O, Ting R. One-Step, Rapid, ^*18*^F–^*19*^F Isotopic Exchange Radiolabeling of Difluoro-dioxaborinins: Substituent Effect on Stability and In Vivo Applications. J Med Chem. 2020;63:12693–12,706. Copyright (2020) American Chemical Society. *Printed with permission from* *Hu* et al.*,* 2020*by Copyright Clearance Center’s Rightslink)*

Many anticancer drugs, antibiotics and natural products have a β-diketone moiety that can be modified into difluoro-dioxaborinin via a β-diketone-boron trifluoride reaction. Several groups have recently reported ^18^F–^19^F Isotopic Exchange (IEX) methods for molecules containing a Si-F or a B-F bond. Recently, a rapid (< 10 min), one-step, R.T. and high yielding (> 80%) SnCl_4_-catalyzed ^18^F–^19^F IEX radiolabeling method has been described (Fig. 4) for introducing ^18^F into various difluoro-dioxaborinins (An et al., 2020). Interestingly, such [^18^F]difluoro-dioxaborinins were produced in high molar activities (270 GBq/μmol) starting from low amount of ^18^F (590 MBq). In aqueous solutions, these molecules eventually undergo multistep solvolysis and convert back to the parent β-diketones after several hours (Fig. 4) allowing for PET and fluorescence imaging. The electronegativity of the substituents was shown to affect the stability and the fluorescence properties of difluoro-dioxaborinin probes. They demonstrated enhanced solvolysis stability with electron-donating substituents at the R1 and R2 positions of difluoro-dioxaborinin 4,5,6 π-systems through hyperconjugation and induced enhanced red-shifted optical properties. This new procedure for ^18^F-radiolabeling a β-diketone-bearing structures has the potential to afford solvolysis-resistant and highly fluorescent novel agents for dual PET/fluorescence imaging modality.

### Impact of ^68^Ga-FAPI-PET/CT imaging on the therapeutic management of primary and recurrent pancreatic ductal adenocarcinomas

#### By Zhibo Liu

This study (Rohrich et al., 2020) describes 19 patients with pancreatic cancer (7 with early-stage primary pancreatic cancer and 12 with progressive/recurrent pancreatic cancer). Three of the patients were visualized with ^68^Ga-FAPI-46, and the remaining patients were visualized with ^68^Ga-FAPI-04. The authors collected and recorded the SUVmax and SUVmean of pancreatic cancer and metastases and compared the change in TNM stage of patients before and after PET examination.

It was concluded that:

1. SUVmax and SUVmean of primary pancreatic cancer lesions, lymph node metastases, and distant organ metastases were significantly higher than those of other normal organs. ^68^Ga-FAPI can detect metastases (lymph nodes, liver, bone) that were more difficult to detect with enhanced CT, and ^68^Ga-FAPI PET/CT changed the TNM staging in 10 patients.
2. The diagnostic ability of FAPI-04 and FAPI-46 performed comparably.
3. The SUV of pancreatic cancer lesions was higher than that of pancreatitis lesions, but the SUV still had overlap.

### Comments

The number of patients included in this paper was small, and the main focus of attention was on the diagnosis of pancreatic cancer. From the conclusion, it can be seen that ^68^Ga-FAPI PET/CT can play an important role in the diagnosis of pancreatic cancer, with better results than clinical conventional enhanced CT with ^18^F-FDG PET/CT.

### Highlight

It is sometimes difficult to distinguish clinical pancreatic cancer from pancreatitis lesions, and some pancreatic cancers are also combined with pancreatitis. In a patient with pancreatic cancer combined with distal pancreatic obstructive pancreatitis, it was found that the SUV of pancreatic cancer lesions decreased insignificantly with prolonged imaging time, while the SUV of pancreatitis lesions decreased more significantly with prolonged imaging time, and the longer the examination time, the greater the difference between pancreatic cancer and pancreatitis lesions. However, this trend was not evident in another patient, so there is still a need to include more patients for relevant studies

### ^18^F-labeled radiotracers for in vivo imaging of DREADD with positron emission tomography

#### By Jan Passchier

**Fig. 5:**
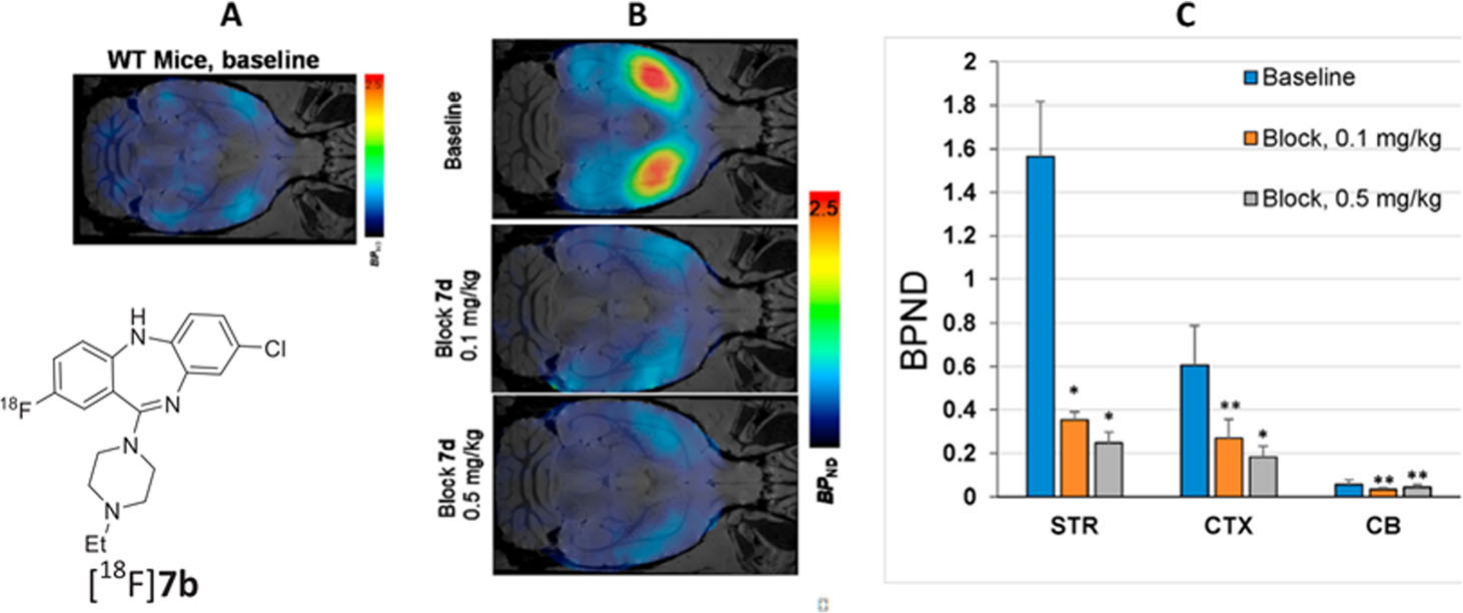
Panel **a**: Transverse PET-[^18^F]**7b** of wild-type mouse; Panel **b**: Transverse PET-[^18^F]**7b** images of tansgenic mice expressing hM3Dq (baseline and dose-escalation blocking); Panel C: Brain regional binding potential (BP_ND_) values in baseline and dose-escalation blocking PET scans with [^18^F]7b in transgenic mice expressing hM3Dq. Abbreviation: STR = striatum; CTX = cortex; CB = cerebellum. Blocker **- 7d**. Statistical analysis: comparison of baseline versus blocking in the same region. *P<0.05: **P>0.05

Adeno associated virus (AAV) capsids have emerged as clinically acceptable vectors for gene therapy. To assess AAV delivery and gene payload transfection after administration, there is a need to delineate both AAV distribution and resulting protein expression in preclinical and early phase clinical trials. For protein expression, the use of designer receptors exclusively activated by designer drugs (DREADDs) can be of particular interest. DREADDs are a series of engineered human muscarinic receptors that respond exclusively to the synthetic ligand clozapine N-oxide (CNO) thereby allowing for spatial and temporal control of G-protein signalling in vivo. As such, these receptors can be both therapeutic and, given its lack of interaction with endogenous ligands and the low mass dose approach of PET imaging, act as low-pharmacological marker to enable quantification of protein expression. Hu et al. (2020) report a fluorine-18 labelled derivative of [^11^C]clozapine with improved imaging utility, [^18^F]7b. High and specific binding of [^18^F]7b was observed in transgenic mice expressing the D1**-**hM3Dq DREADD receptor compared to wild type mice (see Fig. 5, modified from the original to include the structure of [^18^F]7b). While further work is required to optimise imaging approaches, this work highlights the possibility of using PET to support the development of novel gene therapies, especially for CNS applications.

### Selective ^18^F-radiolabeling of peptides with [^18^F]Fluoro-4-(vinylsulfonyl)benzene

#### By Peter J. H. Scott

**Fig. 6:**
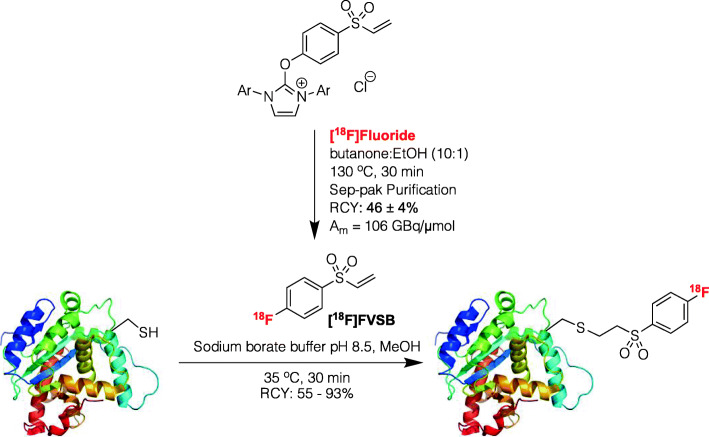
[^18^F]FVSB, a thiol reactive synthon for mild and rapid radiolabeling of peptides. Reprinted (adapted) with permission from Ma G, McDaniel JW, Murphy JM. One-step synthesis of [^18^F]Fluoro-4-(vinylsulfonyl)benzene: a thiol reactive synthon for selective radiofluorination of peptides. Org Lett. 2021;23:530–534. Copyright (2021) American Chemical Society

Radiolabeled peptides are important imaging agents for positron emission tomography and, as such, there is enormous motivation to develop mild and reliable methods for their preparation (Charron et al., 2016). In the context of fluorine-18 radiochemistry it is typical to radiolabel peptides using prosthetic groups (e.g. thiol-reactive synthons such as labeled maleimide derivatives). Despite continued advances, such approaches can still be challenging given unstable precursors and the necessity for both time-consuming azeotropic drying and HPLC purification. To overcome these issues, the Murphy group have capitalized on new methods for late-stage radiofluorination ([^18^F]deoxyfluorination of readily accessible uronium precursors (Figs. 6); Neumann et al., 2016) to develop a one-step synthesis of [^18^F]fluoro-4-(vinylsulfonyl)benzene ([^18^F]FVSB) (Ma et al., 2021). The process does not require azeotropic drying or HPLC purification, and provides [^18^F]FVSB in good radiochemical yields (88–97% (manual) and 63% (preliminary automation) RCY based upon radio-TLC, 46 ± 4% isolated RCY (manual)) and high molar activity (106.2 GBq/μmol). [^18^F]FVSB benefits from the high reactivity of the vinyl sulfone motif for free thiols, allowing formation of stable thioether linkages with peptidic cysteine residues. The team demonstrates proof-of-concept through the facile preparation of several stable, site specific ^18^F-labeled peptide conjugates. Cysteine bioconjugation with [^18^F]FVSB proceeds in aqueous conditions at 35 °C for 30 min. Bombesin, PSMA, RGD and neuromedin B analogues were obtained in 55–93% RCY. Overall, this approach represents an attractive approach for the mild and rapid ^18^F-labeling of a variety of peptides that is amenable to automation.

### Fluorine-18-labelled antibody ligands for PET imaging of amyloid-β in brain

#### By Danielle Vugts

Despite their specificity, antibodies have not been extensively used as therapeutics for neurological disorders. This is mainly because of their low blood-brain-barrier penetration resulting in low drug levels in the brain. This can be overcome via receptor mediated transcytosis, e.g. using the transferrin receptor expressed on endothelial cells. Previously, in mice it was proven that the uptake in the brain of bispecific antibodies consisting of an anti-transferrin and amyloid-β part could be considerably increased and a specific PET signal could be detected using ^124/125^I. In addition. The radiolabeling with the shorter radionuclide fluorine-18 via inverse electron demand Diels-Alder chemistry (Syvanen et al., 2020) offers additional radiolabelling oportunities. Three different ^18^F-labeled tetrazines were prepared with good radiochemical yields. The reaction with the TCO-modified mAbs was less efficient, mainly caused by the low amounts of mAb that were used in the radiolabeling. Despite the fact that the half-life of the mAbs was not ideal for imaging with fluorine-18, one of the ^18^F-labeled mAbs showed a specific uptake when the SUV ratio of frontal cortex over cerebellum was calculated. Ex vivo there was no difference between wildtype and tg-ArcSwe mice. Clearly there is room for improvement and proteins with shorter serum half-life are needed to be able to image them with fluorine-18.

### ^68^Ga -labeled PD-L1 targeted nanobody is available now!

#### By Zhi Yang

Immunotherapy through programmed death 1/programmed death ligand 1 (PD-1/PD-L1) checkpoint blockade has shown impressive clinical outcomes, but not all oncological patients do respond. Many studies have demonstrated that the expression level of PD-L1 in tumors is one of the factors that correlate with the PD-1/PD-L1 checkpoint blockade therapy. Thus, it is essential to analyze the PD-L1 expression in patients prior to treatment, which may avoid the ineffective cure and improve the success rate of immunotherapy. Recently, a novel ^68^Ga-labeled nanobody, named ^68^Ga-NOTA-Nb109, was designed and developed for non-invasive molecular imaging of PD-L1 expression in the melanoma-bearing mice model (Lv et al., 2020). It was generated with high affinity toward PD-L1 in good radiochemical yield. Micro-PET imaging studies on the tumor-bearing models with high, medium, and low PD-L1 expression demonstrated that the tracer could distinctly distinguish different tumors and noninvasively quantify the PD-L1 expression in tumors. Furthermore, it showed the different binding sites to PD-L1 comparing with anti-PD-L1 antibodies. The tracer holds great potential for PD-L1 detection, evaluating the immunotherapeutic effect and optimizing the prescription for PD-1/PD-L1 checkpoint blockade therapy.

### A sound example of useful basic radiochemistry

#### By Adriano Duatti

This paper (Rodnick et al., 2020) describes a nice piece of radiochemistry, but its main virtue is that it was rigorously designed and, therefore, it may serve as a guide for radiochemists. In particular, it addressed a real and persistent critical issue in medical radionuclide production related to the limited supply of the important diagnostic radionuclide gallium-68. Since the potential diagnostic applications of ^68^Ga-radiopharmaceuticals are expected to increase constantly in the near future, the intrinsic drawbacks of the ^68^Ge/^68^Ga generator will become more and more apparent and possibly hamper the rapid introduction of new ^68^Ga-based diagnostic agents into the clinical practice. Again, this is a real and potentially disrupting problem and, therefore, absolutely worthy to be seriously taken into account. In this context, the other significant value of this paper is that the proposed technical solutions did not look for sophisticated and expensive technologies that might get difficult their subsequent application, but rather they fully exploited existing technologies and equipment. Obviously, the results described in the paper do not allow yet to definitively address all experimental and technical issues, but the whole scientific approach undoubtedly constitutes a remarkable example of good radiochemistry serving the real and actual needs of nuclear imaging.

### Synthesis, radiolabelling and pre-clinical evaluation of [^44^Sc]Sc-AAZTA conjugate PSMA inhibitor, a new tracer for high-efficiency imaging of prostate cancer

#### By Silvio Aime

Scandium-44 continues to be under intense scrutiny as a potential radionuclide with favorable characteristics for PET applications. Its half-life of 3.97 h matches the rate of some slow targeting processes involving peptides and antibody fragments. Furthermore, scandium-44 can be considered an appealing candidate to develop new theranostic strategies when combined with a radionuclide having radiotherapeutic properties such as the β-emitting scandium-47. A new PSMA inhibitor derivative imaging probe (B28110) conjugated with AAZTA ligand (Ghiani et al., 2021) has shown superior properties when compared with the corresponding commercially available DOTA-based PSMA-617 (same targeting ligand) for the PET diagnosis of prostate cancer. AAZTA (a heptadentate aminopolycarboxylate ligand with a 1,4-diazepine scaffold, easy to synthetize and to conjugate, thoroughly studied as ligand of Gd^3+^ ions for MRI applications) confirmed its outstanding ability to chelate scandium-44 radionuclide under mild experimental conditions (RCY > 95%, at room temperature, pH 4, with short reaction time (5 min)). In vivo PET/MRI imaging on LNCaP tumor-bearing mice showed high tracer accumulation in the tumor region as early as 20 min post injection. Ex vivo bio-distribution studies confirmed that the accumulation of ^44^Sc-PSMA-617 was two-fold lower than that of the newly developed [^44^Sc]Sc-B28110 probe. The good performance of [^44^Sc]Sc-B28110 to detect prostate cancer cells was also demonstrated in respect to the related gallium-68 analogue ([^68^Ga]Ga-B28110) bearing the same PSMA targeting inhibitor.

### [^11^C]carbonyl Difluoride-a new and highly efficient [^11^C]carbonyl group transfer agent

#### By guy Bormans

Carbon-11 remains a valuable radionuclide for positron emission tomography. The exposure to ionising radiation of subjects injected with a ^11^C-radiopharmaceutical (the absorbed radiation dose) will be limited to 5 μSv/MBq thanks to the short half-life (20.4 min) of carbon-11 (Zanotti-Fregonara et al., 2021). This allows performing multiple PET scans in the same subject which is especially interesting to study e.g. quantitative brain pharmacodynamics of novel drug candidates as target occupancy can be quantified as function of drug dose and/or time after drug administration in the same subject. Carbon-11 allows to perform isotopic labelling of a target molecule so that the biological characteristics of the ^11^C-labelled PET tracer are identical to those of the stable ^12^C isotopologue.

The short half-life however also limits the maximal PET scan duration, imposing restraints on the pharmacokinetics of ^11^C-labelled PET tracers. From a radiochemistry/radiopharmaceutical point of view, the short half-life renders the production of ^11^C-radiopharmaceuticals quite challenging as only fast and high-yield reactions and procedures can be employed and only a limited number of subjects can be injected from one production batch.

**Fig. 7:**
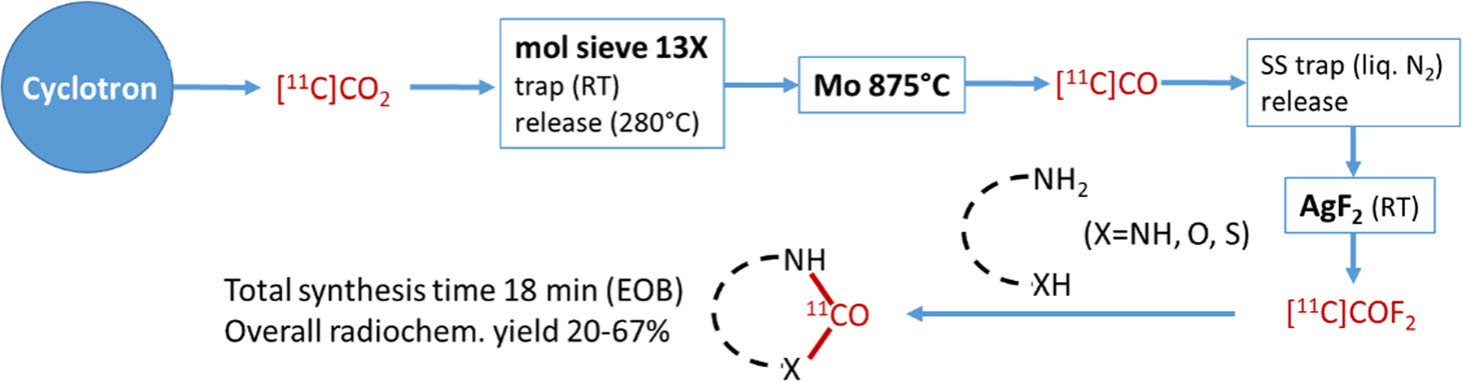
Total synthesis time 18 min (EOB). Overall radiochem yield 20-67%

In order to expand carbon-11 radiochemical space it is important to develop novel ^11^C-synthons that can be produced with simple, reliable, high-yield and robust procedures. Jacobsson and colleagues describe the efficient radiosynthesis of [^11^C]carbonyl difluoride starting from [^11^C]CO_2_ using a two-step gas-phase procedure (Jakobsson et al., 2020). In the first instance, [^11^C]CO_2_ harvested from the cyclotron target is converted by a well–known procedure to [^11^C]CO by passage over a heated (875 °C) column filled with molybdenum (See Fig. 7). [^11^C]CO is then cryogenically trapped and released with a stream of helium over a stainless steel column at room temperature filled with AgF_2_ to quantitatively convert [^11^C]CO to [^11^C]COF_2_ which is bubbled through the carbonylation reaction mixture. Various short (5 min) room temperature carbonylation reactions starting from diamines, amino-alcohol and aminothiols demonstrated the versatility and robustness of the methodology providing excellent radiochemical yields (30–96%) with relatively small mass amounts (0.25 mg) of precursor even in the presence of atmospheric moisture.

The method can be fully automated using similar equipment as used for gas phase production of [^11^C]CH_3_I/OTf and thus has potential to be adopted widely in carbon-11 radiochemistry labs.

### First human trials of the selective HDAC6 tracer [^18^F]EKZ-001

#### By Jun Toyohara

**Fig. 8:**
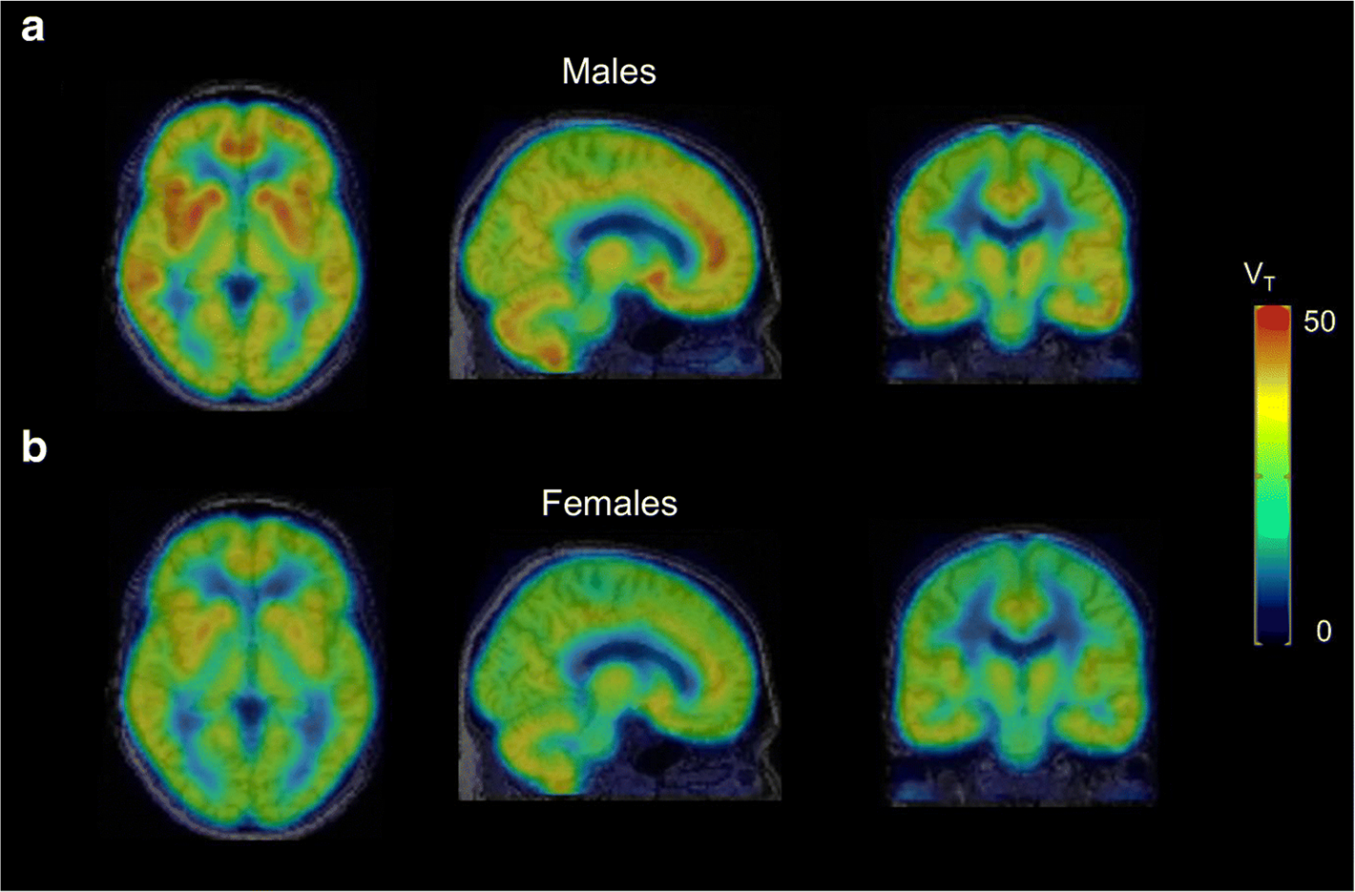
Average parametric Logan graphical analysis (LGA) V_T_ datasets for a 120-min [^18^F]EKZ-001 PET scan (*n* = 6 subjects per group, cohorts for radiotracer kinetic modeling, test-retest and inter-subject variability in brain, only the first scan was considered for subjects with 2 scans) for males **a** and females **b**. This figure was obtained from Koole et al. (2020) which was licensed under a Creative Commons License https://creativecommons.org/licences/by/4.0/

Histone deacetylase 6 (HDAC6) is a cytoplasmic enzyme that removes acetyl groups from multiple substrates, thereby influencing diverse cellular functions. Several radiotracers for imaging HDAC6 in the brain have been developed, but the design of brain-penetrating HDAC6 ligands remains challenging due to the ready ionization of hydroxamic acid groups necessary for chelate zinc ions in the catalytic site of HDAC6. Recently, the HDAC6-selective adamantyl group-conjugated hydroxamic acid radiotracer [^18^F]EKZ-001 (also known as [^18^F]bavarostat) was developed and was shown to penetrate the brain efficiently (Strebl et al. 2016). Following their successful preclinical validation studies (Celen et al. 2020), Koole and colleagues conducted the first human PET study to investigate the utility of [^18^F]EKZ-001 in dosimetry, as well as for kinetic modeling of the brain (Koole et al. 2020). [^18^F]EKZ-001 was found to be safe and the amount of radioactivity required for imaging was within the acceptable effective dose range for initial clinical trials. The appropriate brain kinetic model for [^18^F]EKZ-001 was the two-tissue compartment model (2TCM), and Logan graphical analysis (LGA) provided limited bias and better test-retest variability and reliability than 2TCM. The regional total distribution volume (V_T_) values were relatively homogeneous, with the highest values observed for the hippocampus and entorhinal cortex. The acquisition time was reduced to a 1 h scan (0–60 min), followed by a 30 min break, and then a 30 min scan (90–120 min). Interestingly, males showed higher LGA V_T_ values across the cortical and subcortical brain regions compared to females (Fig. 8), suggesting that the HDAC6 gene is located on the X-chromosome.

## Conclusions

Trends in radiochemistry and radiopharmacy are highlighted demonstrating the progress in the research field being the scope of EJNMMI Radiopharmacy and Chemistry.

## Data Availability

Datasets mentioned in this article can be found in the cited articles.

## Abbreviations

AAZTA: 1,4-bis(carboxymethyl)-6-[bis(carboxymethyl)]amino-6-methylperhydro-1,4-diazepine
AAV: Adeno associated virus
AKT: Protein kinase B
BBN: Bombesin
DNA: Deoxy Ribonucleic Acid
DOPA: Di-hydroxy phenylalanine
DREADD: Designer receptors exclusively activated by designer drugs
FAPI: Fibroblast activation protein inhibitors
GRPR: Gastrin Releasing Peptide Receptor
GMP: Good Manufacturing Practice
HDAC: Histone deacetylase
HPLC: High Performance Liquid Chromatography
IEX: Isotopic Exchange
mCRPC: Metastatic castration-resistant prostate cancer
MNP: Melanin nanoparticles
MRI: Magnetic Resonance Imaging
PET: Positron Emission Tomography
PI3K: Phosphoinositide 3-kinase
PD-L1: Programmed death-ligand 1
PSMA: Prostate Specific Membrane Antigen
PTC: Phase Transfer Catalyst
RCC: Radiochemical Conversion
RCY: Radiochemical yield
RGD: Arginylglycylaspartic acid
SFB: Succinimidyl 4-[^18^F]fluorobenzoate
TBAOTs: Tetrabutylammonium tosylate
TCO: Trans Cyclo Octene
TRT: Targeted Radionuclide Therapy
